# Predicting extubation failure in patients with cardiorespiratory decompensation: development and validation of an interpretable machine learning–based risk calculator

**DOI:** 10.3389/fcvm.2026.1862117

**Published:** 2026-08-24

**Authors:** Ting Zhang, Tianqi Lu, Sha Yang

**Affiliations:** The First People’s Hospital of Lin'an District, Hangzhou, Zhejiang, China

**Keywords:** clinical translation, prognostic prediction, respiratory failure with cardiac dysfunction, SHapley additive exPlanations (SHAP) value analysis, visualized decision support

## Abstract

**Objective:**

This study aimed to develop an interpretable machine learning model for predicting extubation failure in mechanically ventilated patients with combined respiratory failure and cardiac dysfunction.

**Methods:**

A retrospective cohort of 350 patients receiving invasive mechanical ventilation between January 2022 and January 2024 was analyzed. After rigorous data preprocessing ≤5% missing values were imputed using mean, median, or mode based on distribution type. The dataset was partitioned into training (*n* = 245) and testing (*n* = 105) sets at 7:3 ratio. Feature selection and hyperparameter tuning were synchronously optimized using an Improved Snow Geese Algorithm (ISGA)-driven Automated machine learning (AutoML) framework. Six supervised models [Logistic Regression (LR), Support vector machine (SVM), Adaptive Boosting (AdaBoost), eXtreme Gradient Boosting (XGBoost), Light Gradient Boosting Machine (LightGBM), AutoML] were evaluated using six classification metrics [Accuracy, Sensitivity, Specificity, F1-score, Area Under the Receiver Operating Characteristic Curve (ROC-AUC), Area Under the Precision-Recall Curve (PR-AUC)] with five-fold cross-validation. Calibration curves and Brier scores assessed reliability, while SHAP analysis provided interpretability of predictors.

**Results:**

The ISGA-optimized AutoML framework (converging to LightGBM as the optimal algorithm) demonstrated superior performance on the independent test set, achieving a ROC-AUC of 0.9289 and a PR-AUC of 0.8892. Decision curve analysis confirmed clinical utility across threshold probabilities of 0.3–0.7, outperforming treat-all and treat-none strategies, and the calibration curve yielded a low Brier score of 0.114. Key predictors identified included brain Natriuretic Peptide (BNP), lactate, left ventricular ejection fraction (LVEF), troponin, and central venous pressure (CVP).

**Conclusion:**

The interpretable prediction tool accurately stratifies extubation failure risk using clinically available parameters, offering a prototype tool for personalized ventilation management.

## 1 Introduction

Cardiorespiratory diseases represent leading global causes of mortality and disability (1, 2). Respiratory failure, a critical clinical syndrome, indicates impaired pulmonary ventilation and/or gas exchange, compromising the body's oxygen supply and carbon dioxide elimination. Its etiology encompasses pulmonary pathologies [e.g., pneumonia, chronic obstructive pulmonary disease (COPD), interstitial lung disease, acute respiratory distress syndrome (ARDS)] and extrapulmonary triggers (e.g., heart failure) (3, 4). Cardiac dysfunction refers to clinical syndromes involving impaired ventricular filling or ejection due to structural or functional heart diseases. Significant pathophysiological interactions exist between these conditions: severe left heart failure elevates pulmonary venous pressure, precipitating cardiogenic pulmonary edema that induces or exacerbates respiratory failure; conversely, chronic/acute respiratory diseases causing hypoxemia, hypercapnia, and pulmonary hypertension increase right ventricular afterload, potentially culminating in right or biventricular failure (5–7). The coexistence of respiratory failure and cardiac dysfunction signifies cardiopulmonary decompensation. Patients exhibit dire prognosis with rapid deterioration, including refractory hypoxemia, hemodynamic instability, heightened multi-organ dysfunction risks, persistently elevated mortality, and frequent readmissions, imposing substantial public health and resource burdens (8, 9).

Accurate and timely prognostic assessment in such critically ill patients is essential for clinical decision-making, particularly for predicting extubation failure in mechanically ventilated individuals. Effective stratification aids in identifying high-risk individuals, optimizing resource allocation, guiding personalized therapy (e.g., advanced life support, pharmacotherapy), and facilitating precise communication with patients/families regarding realistic treatment goals (10, 11). Nonetheless, while APACHE II and SOFA scores retain clinical utility for mortality risk estimation through composite weighted markers, they are fundamentally designed to quantify severity rather than predict specific outcomes like extubation failure (12, 13). Primary limitations include: (1) Multi-factor interactivity: Prognosis depends not only on baseline cardiorespiratory function but is dynamically influenced by demographics (e.g., age), comorbidities (e.g., diabetes, renal impairment), laboratory markers (e.g., inflammatory indices, blood gas analysis, cardiac biomarkers), real-time vital signs, and respiratory support intensity. These variables exhibit complex nonlinear interactions. Traditional statistical constraints: Conventional linear models (e.g., logistic/Cox regression) assume linear variable-outcome relationships and inadequately address multicollinearity, feature interactions, or high-dimensional data. They fail to fully uncover hidden patterns and prognostic contributions of key variables when handling real-world complex data structures with nonlinear dependencies (14, 15). Given this complexity, supervised machine learning provides a viable approach for directly predicting extubation failure by leveraging high-dimensional data and capturing nonlinear relationships.

In recent years, machine learning (ML)—a core branch of artificial intelligence—has demonstrated substantial potential in medical research and clinical practice through its powerful data analysis capabilities, offering novel approaches to address the aforementioned challenges. ML algorithms autonomously extract patterns from massive, high-dimensional, heterogeneous clinical data. Their core strengths include handling complex structures and high-dimensional data, robust pattern recognition and prediction capabilities, potential for individualized prediction, and continuous learning aptitude (16). Particularly in critical care, cardiovascular diseases, and respiratory disorders, ML models have flourished in mortality prediction, complication risk early-warning, disease stratification, and optimized therapeutic decision support, with proven significant improvements in predictive accuracy (17, 18). However, a critical bottleneck hindering the widespread clinical adoption of ML technology is its “black-box nature”. Although many high-performance ML models (especially deep learning) excel in predictive accuracy, their internal decision-making logic is often opaque or incomprehensible. Clinicians cannot intuitively discern the rationale behind a specific prediction nor identify which key features drive the outcome. This lack of interpretability substantially limits clinician trust and acceptance, impedes validation of biological plausibility, obstructs discovery of new knowledge, and hampers the ability to question or intervene in individual patient predictions when necessary.

Given the critical importance of prognosticating extubation failure in mechanically ventilated patients with combined respiratory failure and cardiac dysfunction, the inadequacies of existing assessment tools, and the advanced capabilities of ML for complex pattern recognition, this study aims to develop and validate a supervised machine learning–based prognostic prediction framework. The core objective is to create an interpretable prediction model for extubation failure, leveraging explainable AI techniques, and translate it into a bedside clinical decision support tool for personalized risk assessment and intervention.

## 2 Methods

### 2.1 Study population and data collection

This retrospective study enrolled patients with combined respiratory failure and cardiac dysfunction admitted to The First People's Hospital of Lin'an District, Hangzhou, between January 2022 and January 2024. A total of 350 subjects were included after applying predefined criteria. Ethical approval was obtained from the hospital's Institutional Review Board (Approval No.: 2025-35), with waived informed consent due to the retrospective design.

Inclusion criteria: (1) Diagnosis of both respiratory failure [defined as impaired ventilation/gas exchange causing hypoxemia or hypercapnia (19)] and cardiac dysfunction [ventricular filling/ejection impairment, e.g., reduced left ventricular ejection fraction (LVEF) or New York Heart Association (NYHA) class III/IV (20)]; (2) Receipt of invasive mechanical ventilation during hospitalization; (3) Complete clinical documentation.

Exclusion criteria: (1) Age <18 years; (2) > 5% missing data; (3) Isolated respiratory failure or cardiac dysfunction; (4) Severe cognitive/psychiatric disorders; (5) Active malignancy.

Data were extracted structurally from electronic medical records and verified by a trained researcher. Collected variables included: (1) Demographics: Age, sex, BMI; (2) Comorbidities: COPD, hypertension, diabetes, chronic kidney disease; (3) Admission vitals: Heart rate, blood pressure, respiratory rate, SpO_2_; (4) 24-h lab parameters: Lactate, C-reactive protein (CRP), brain Natriuretic Peptide (BNP), troponin, arterial blood gases; (5) Cardiac function: NYHA class, LVEF, central venous pressure (CVP); (6) Therapy: Mechanical ventilation mode, medications. Outcome definition: Extubation failure. For terminological consistency, we use the term extubation failure throughout this manuscript to refer to the primary outcome. Extubation failure was defined as the composite of reintubation or unplanned rescue non-invasive ventilation (NIV) within 48 h following planned extubation (21). The following events were excluded from the failure definition: (1) Planned/prophylactic post-extubation NIV: In patients with known cardiac dysfunction or COPD, scheduled initiation of NIV immediately after extubation as a preventive strategy was not considered a failure. (2) Reintubation for non-respiratory indications: Surgical re-exploration, airway protection for neurological deterioration (e.g., stroke, altered consciousness), or procedures unrelated to respiratory decompensation. (3) Death unrelated to respiratory failure: Fatal events clearly attributable to non-respiratory causes (e.g., terminal malignancy progression, catastrophic intracranial hemorrhage) were censored rather than counted as extubation failure. High-flow nasal cannula (HFNC) was not used as a rescue therapy for extubation failure in our study population and therefore was not part of the failure definition. Death within 48 h that could not be clearly attributed to a non-respiratory cause was conservatively counted as extubation failure. The primary prediction time point of this model is the moment of clinical extubation decision-making. All predictor variables were required to be available prior to this decision. Demographic data, comorbidities, and admission vital signs were recorded at hospital admission. The 24-h laboratory parameters (including lactate, CRP, BNP, troponin, and arterial blood gases) were defined as the most recent measurements within the 24 h immediately preceding the extubation decision. To handle repeated measurements within this 24-h window, we selected the value closest to the time of the extubation decision (i.e., the last available record before extubation), as it best reflects the patient's physiological status at the point of risk assessment. No multiple-imputation or averaging of repeated measures was performed; if only a single measurement existed within the window, that value was used. This approach avoids artificial manipulation of temporal dynamics and ensures that the predictor values are clinically congruent with the intended use of the model at the bedside.

In this study, a missing rate ≤5% was set as the data completeness control standard, but the proportion of missing data was not used as the sole basis for selecting imputation methods. Patients with an overall missing proportion >5% in candidate fields were excluded at enrollment; meanwhile, candidate variables with an overall missing rate >5% were not entered into model development. Among the 350 patients finally included, 287 (82.0%) were complete cases, and the remaining 63 (18.0%) had a small amount of sporadic missing data. The missing rate of the included variables ranged from 0.3% to 4.5%, and the missing rates of the core predictive factors in the final model were all ≤1.5%. Considering that whether certain laboratory tests were performed might be related to the patient's already observed condition, vital signs, and clinical diagnosis and treatment process, this study assumed the missing data to be missing at random (MAR), meaning that after controlling for the observed clinical information, the probability of missingness no longer depended on unobserved values. The MAR assumption cannot be fully validated solely through observed data, thus the possibility of non-random missingness still cannot be completely ruled out. For continuous variables with a normal distribution, mean imputation was used; for continuous variables with a skewed distribution, median imputation was used; and for categorical variables, mode imputation was used. To avoid information leakage, in each fold of the five-fold cross-validation, the imputation parameters were calculated based only on the training subset of that fold and then applied to both the training subset and the validation subset of that fold. After the final model was determined, the imputation parameters were re-estimated based on the complete training set and applied to the independent test set. Information from the test set did not participate in the estimation of imputation parameters.

### 2.2 Automated machine learning

We proposed an Improved Snow Geese Algorithm (ISGA)-driven Automated Machine Learning (AutoML) framework for dual objectives: screening key features and optimizing hyperparameters in extubation failure risk prediction. We enhance Snow Geese Algorithm (SGA) (22) to ISGA by: (1) using chaotic mapping to optimize initial population distribution for enhanced spatial ergodicity; (2) incorporating dynamic Lévy flight step sizes to balance search efficiency in exploration-exploitation stages. ISGA performance was validated via CEC2022 benchmarks. The parameter configuration of the ISGA algorithm is detailed in Supplementary File 1 Table S1.

The ISGA-AutoML framework performs synchronous optimization within a five-fold cross-validation framework (pseudocode provided in Supplementary File 2). The original feature space contains 20 candidate variables spanning six categories: demographics, comorbidities, admission vital signs, 24-h laboratory tests, cardiac function, and treatment regimens. The encoding vector for each individual consists of three components: a feature mask (20-dimensional binary encoding), a base learner index (integer encoding from 1 to 6), and a hyperparameter vector (mixed continuous/integer encoding, up to 9 dimensions). The candidate base learner pool includes six classification models: Logistic Regression (LR), Support Vector Machine (SVM), Decision Tree (DT), AdaBoost, XGBoost, and LightGBM. In each fold of cross-validation, the following steps are sequentially performed on the training subset: (1) feature selection—optimizing the feature mask in discrete space; (2) base learner selection—selecting the optimal algorithm from the six candidate models; (3) hyperparameter tuning—optimizing the hyperparameter configuration of the selected model in continuous/integer spaces (the hyperparameter search ranges are provided in Supplementary File 1 Table S2). The performance is evaluated on the validation subset using ROC-AUC as the fitness function. The features selected in each fold are ranked by frequency to determine the final feature subset, the optimal hyperparameters are taken as the mean across folds, and the base learner is the model selected in the majority of folds. It should be noted that this framework is not an ensemble learning or meta-learning pipeline, but rather an automated single-model selection and hyperparameter tuning system based on swarm intelligence optimization. The optimization population size is 30, the maximum number of iterations is 50, and the process is independently run 10 times, with the configuration corresponding to the best fitness value selected as the final solution.

Sample size justification: The sample size of this study was determined by consecutive enrollment within the prespecified study period. Sample adequacy was assessed with reference to the 10 EPV (Events Per Variable) criterion proposed by Peduzzi et al., which requires at least 10 positive outcome events per predictor variable during predictive model development to ensure model stability. The training set comprised 245 patients, of whom 118 (48.2%) experienced extubation failure. According to the 10 EPV standard, this can stably support a maximum of approximately 11–12 predictor variables simultaneously in the model. In this study, the ISGA-AutoML framework implements feature selection through feature mask optimization within each fold of cross-validation. The number of features entering the base learner in each fitness evaluation is the candidate subset during the optimization process, and the maximum number of selected features in the feature mask is set to no more than 11 according to the 10 EPV criterion, thereby ensuring that each model evaluation during the ISGA search process satisfies the statistical stability requirement of EPV ≥ 10.

**Figure 1:**
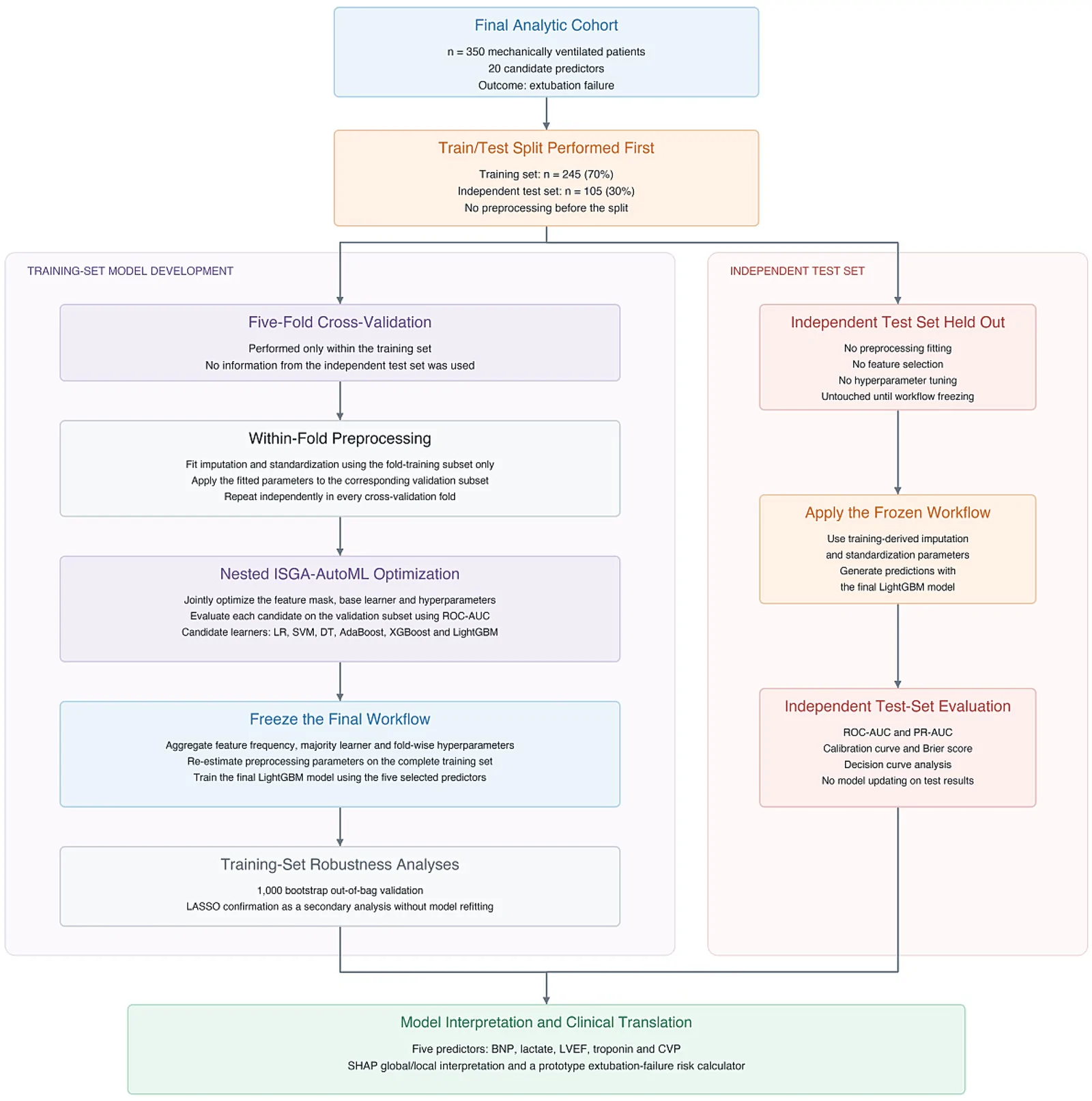
Technical workflow diagram.

The complete analysis workflow was designed as follows to minimize data leakage: (1) The data were divided into a training set and an independent test set at a 7:3 ratio; (2) five-fold cross-validation was implemented on the training set, and in each fold, the missing value imputation parameters and standardization parameters were estimated solely based on the training subset of that fold and then applied to the corresponding validation subset; (3) within each training subset, ISGA was used to simultaneously perform feature selection, base learner selection, and hyperparameter optimization, and fitness was calculated on the validation subset; (4) the results from all folds were combined to determine the final features, base learner, and hyperparameters; (5) the imputation and standardization parameters were re-estimated using the complete training set, and the final model was trained; (6) all preprocessing parameters obtained from the training set were applied to the independent test set to complete the final performance evaluation. Throughout the entire process, no information from the test set was used for preprocessing, feature selection, or parameter optimization. All implementations were performed using MATLAB 2024a. The workflow is shown in Figure 1.

### 2.3 Evaluation metrics

A multidimensional evaluation framework was established: (1) Classification performance: Assessed using six core metrics for prognostic prediction models: Accuracy (ACC), Sensitivity (SEN), Specificity (SPE), F1-score (harmonic mean of precision and recall), Area Under the Receiver Operating Characteristic Curve (ROC-AUC), and Area Under the Precision-Recall Curve (PR-AUC). These metrics systematically evaluated model discrimination; (2) Calibration performance: Evaluated using calibration curves and the Brier score (lower values indicate higher prediction accuracy); (3) Clinical utility: Quantified using Decision Curve Analysis (DCA), calculating the Net Benefit (NB) across threshold probabilities. The NB is defined as:

$$NB=\frac{TP}{N}-\frac{FP}{N}\times \frac{\;p_{t}}{1-p_{t}}$$

where TP is true positives, FP is false positives, N is the total sample size, and pt is the risk threshold. The model's effective clinical decision-making range was validated by comparing NB against treat-all and treat-none strategies.

To quantify the uncertainty of performance metrics and provide resampling-based internal validation, this study performed 1,000 Bootstrap resampling iterations on the training set (*n* = 245). In each iteration, an equal number of samples were randomly drawn with replacement from the training set to form a Bootstrap sample, on which the optimal model (LightGBM) was retrained, and evaluation was conducted on out-of-bag (OOB) samples (i.e., samples not drawn). Through 1,000 iterations, the 95% confidence intervals for each performance metric (ROC-AUC, PR-AUC, ACC, SEN, SPE, PRE, F1, Brier Score) were calculated using the percentile method (the 2.5th and 97.5th percentiles). This approach simultaneously achieved the two objectives of internal validation and uncertainty quantification.

To assess the impact of missing value handling strategies on model results, two sensitivity analyses were conducted. The first was a complete case analysis, including only patients with no missing values in all candidate variables. The second employed the Multiple Imputation by Chained Equations (MICE) method to generate 20 imputed datasets, and model training was completed separately using the same final features, base learner, and hyperparameters. The predicted probabilities on the test set were pooled across the 20 imputed datasets. The main analysis, complete case analysis, and multiple imputation analysis all reported the test set ROC-AUC, PR-AUC, and Brier score; the 95% confidence intervals were obtained through 1,000 Bootstrap resampling iterations. If the three methods yielded consistent effect directions and their confidence intervals overlapped substantially, the model results were considered robust to the missing value handling strategy.

### 2.4 Interpretability analysis

After initial feature selection via the AutoML framework, LASSO regression was applied to validate the robustness of the selected features. Subsequently, the SHapley Additive exPlanations (SHAP) model was used to analyze feature clinical plausibility. The specific workflow comprised: (1) AutoML feature pre-screening: Utilizing predefined search spaces and optimization objectives, the AutoML algorithm automatically identified the feature subset most significantly associated with prognosis; (2) LASSO feature validation: LASSO regression was applied to the AutoML-selected subset. Its sparsity and stability were verified through the regularization constraint mechanism, ensuring feature resistance to overfitting. Differences between LASSO-selected and AutoML-selected features were compared; (3) SHAP interpretability analysis: By calculating Shapley values for each feature, quantitative attribution of feature contributions to individual predictions was achieved; summary plots were comprehensively used to display global feature importance rankings, while waterfall plots, decision path plots, and force plots were employed to visually illustrate the interpretation process of specific prediction cases, thereby systematically revealing the model's intrinsic decision-making logic.

### 2.5 Interactive tool development

For clinical implementation, the App Designer module in MATLAB 2024a was used to develop a prototype clinical decision support tool. This software integrates the final optimized machine learning model to provide clinicians with an intuitive, user-friendly interface for assessing the risk of extubation failure.

### 2.6 Statistical analysis

Data were uniformly imported into the SPSS 26.0 statistical platform. Continuous variables conforming to a normal distribution are presented as mean ± standard deviation (x¯ ± s), while non-normally distributed continuous variables are presented as median with interquartile range (M [Q1, Q3]). Categorical variables are presented as frequency and percentage (n %). For inter-group comparisons: Continuous variables underwent normality testing; if both groups conformed to normality, an independent samples t-test was used; otherwise, the Mann–Whitney U test was applied. Categorical variable comparisons employed Pearson's chi-square test. Statistical significance was determined using *P*-values (two-tailed test, significance threshold *α*=0.05). Results are structured in tables for presentation.

## 3 Results

### 3.1 Baseline characteristics of study population

A total of 350 patients initially receiving invasive mechanical ventilation were included and divided into a training set (245 patients) and a test set (105 patients) in a 7:3 ratio. There were no statistically significant differences between the training and test sets in age (74.44 ± 13.35 years vs. 73.31 ± 13.75 years; both *P* > 0.05), body mass index (BMI), blood pressure, left ventricular ejection fraction (LVEF), central venous pressure (CVP), laboratory indicators (BNP, troponin, lactate, CRP), heart rate, respiratory rate, SpO_2_, PCO_2_, or initial fraction of inspired oxygen (FiO_2_) (all *P* > 0.05). The distribution of sex, NYHA functional class, comorbidities [COPD, hypertension, diabetes, chronic kidney disease (CKD) stage ≥3], and usage of diuretics or vasoactive medications were also similar between the two groups (all *P* > 0.05). The incidence of extubation failure was 48.2% (118 cases) in the training set and 43.8% (46 cases) in the test set, with no significant inter-group difference (*P* = 0.454). In summary, the training and test sets were comparable in major demographic characteristics, cardiac/respiratory indicators, comorbidities, and initial ventilation-related variables, meeting the baseline equilibrium requirements for subsequent modeling and external validation. See Table 1.

**Table 1:** Comparison between training and test sets.

Variable	Training set (*n* = 245)	Test set (*n* = 105)	Statistic	*P*-value
Age (years), Mean ± SD	74.44 ± 13.35	73.31 ± 13.75	*t* = 0.706	0.481
BMI (kg/m^2^), Mean ± SD	23.32 ± 4.28	23.06 ± 3.93	*t* = 0.556	0.579
Systolic BP (mmHg), Mean ± SD	128.14 ± 26.14	126.80 ± 28.21	*t* = 0.416	0.678
Diastolic BP (mmHg), Mean ± SD	76.27 ± 23.42	74.42 ± 24.12	*t* = 0.663	0.508
LVEF (%), Mean ± SD	46.50 ± 11.62	47.56 ± 10.73	*t* = −0.822	0.412
CVP (cmH_2_O), Mean ± SD	11.12 ± 4.29	10.85 ± 4.02	*t* = 0.546	0.586
BNP (pg/mL), M (Q_1_, Q_3_)	324 (100, 754)	388 (127, 672)	*Z* = −0.318	0.751
Troponin (μg/L), M (Q_1_, Q_3_)	0.04 (0.01, 0.13)	0.06 (0.02, 0.13)	*Z* = −0.916	0.36
Lactate (mmol/L), M (Q_1_, Q_3_)	1.38 (0.92, 2.23)	1.34 (1.02, 2.32)	*Z* = −0.533	0.594
CRP (mg/L), M (Q_1_, Q_3_)	5.21 (1.45, 16.99)	4.06 (1.11, 24.74)	*Z* = 0.409	0.683
Heart rate (bpm), M (Q_1_, Q_3_)	95 (76, 110)	93 (73, 112)	*Z* = 0.150	0.881
Respiratory rate (breaths/min), M (Q_1_, Q_3_)	17 (14, 21)	17 (14, 22)	*Z* = −1.655	0.097
SpO_2_ (%), M (Q_1_, Q_3_)	97.08 (92.41, 100.00)	95.92 (91.29, 100.00)	*Z* = 1.269	0.197
PCO_2_ (mmHg), M (Q_1_, Q_3_)	34.42 (27.05, 42.63)	36.24 (30.24, 45.38)	*Z* = −1.405	0.16
Initial FiO_2_ (%), M (Q_1_, Q_3_)	40 (40, 41)	40 (40, 42)	*Z* = −0.587	0.53
Sex, *n* (%)				
Male	157 (64.08)	67 (63.81)	*χ*^2^ = 0.0024	0.961
Female	88 (35.92)	38 (36.19)		
NYHA class, *n* (%)				
Class 1	51 (20.82)	23 (21.90)	*χ*^2^ = 0.253	0.969
Class 2	73 (29.80)	32 (30.48)		
Class 3	76 (31.02)	33 (31.43)		
Class 4	45 (18.37)	17 (16.19)		
COPD, *n* (%)				
No	117 (47.76)	47 (44.76)	*χ*^2^ = 0.2644	0.607
Yes	128 (52.24)	58 (55.24)		
Hypertension, *n* (%)				
No	75 (30.61)	31 (29.52)	*χ*^2^ = 0.0412	0.839
Yes	170 (69.39)	74 (70.48)		
Diabetes, *n* (%)				
No	189 (77.14)	84 (80.00)	*χ*^2^ = 0.3497	0.554
Yes	56 (22.86)	21 (20.00)		
CKD (stage ≥3), *n* (%)				
No	185 (75.51)	78 (74.29)	*χ*^2^ = 0.0590	0.808
Yes	60 (24.49)	27 (25.71)		
Diuretic use, *n* (%)				
No	102 (41.63)	43 (40.95)	*χ*^2^ = 0.0140	0.906
Yes	143 (58.37)	62 (59.05)		
Vasopressor use, *n* (%)				
No	87 (35.51)	33 (31.43)	*χ*^2^ = 0.5435	0.461
Yes	158 (64.49)	72 (68.57)		
Extubation outcome, *n* (%)				
Extubation failure	118 (48.16)	46 (43.81)	*χ*^2^ = 0.5595	0.454
Extubation success	127 (51.84)	59 (56.19)		

### 3.2 Model training performance

**Figure 2:**
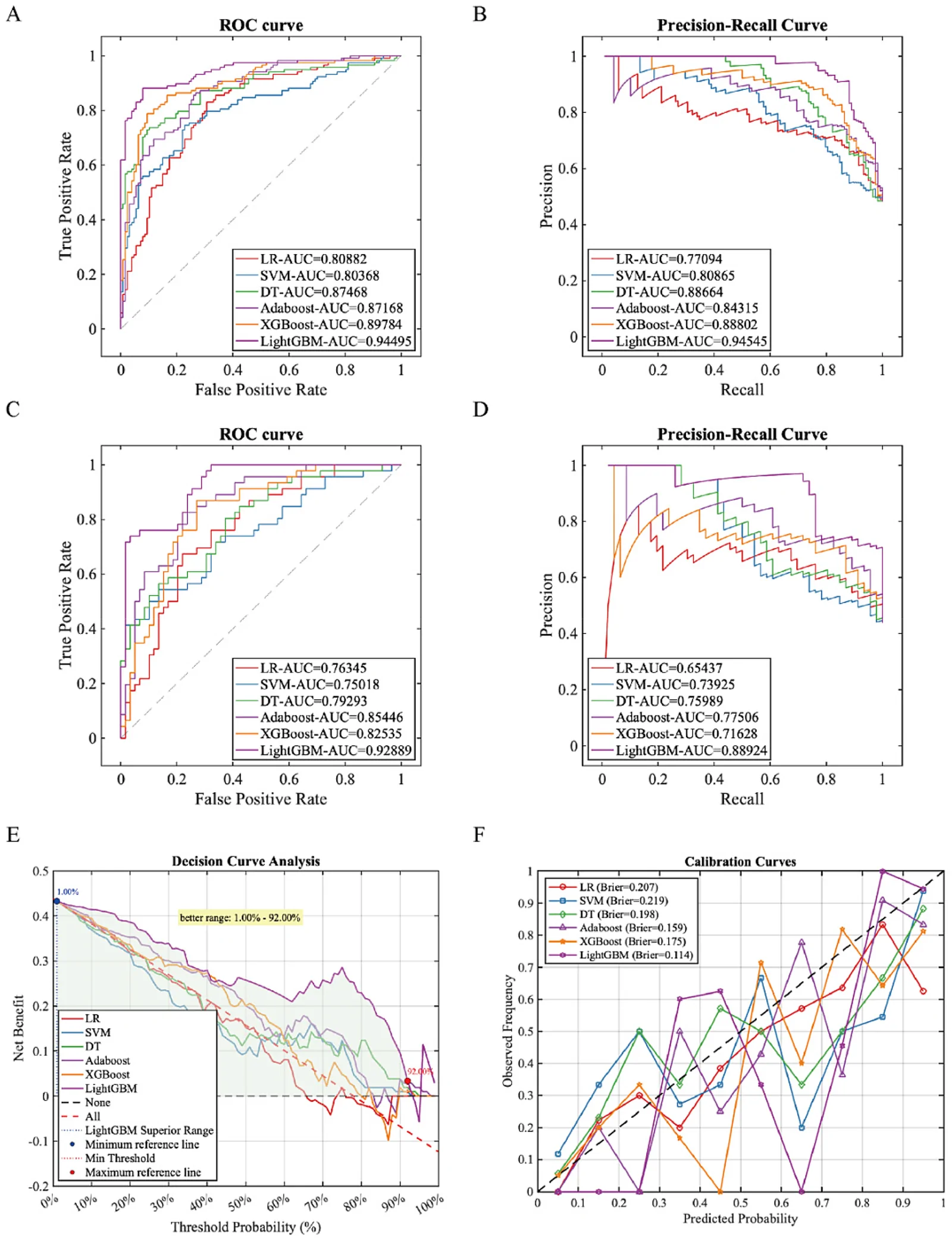
Model training and validation performance. **(A)** Training set ROC curve; **(B)** Training set PR curve; **(C)** Testing set ROC curve; **(D)** Testing set PR curve; **(E)** Testing set DCA curve; **(F)** Testing set calibration curve.

Six machine learning models were trained using these five features. The AutoML model (converging to LightGBM) demonstrated superior overall performance, achieving a training ROC-AUC of 0.9445 and PR-AUC of 0.9455, with an F1-score of 0.8966 highlighting exceptional precision-recall balance (Table 2; Figures 2A,B). Notably, its F1-score (0.8966) highlighted exceptional precision-recall balance, underscoring high clinical utility. AutoML ultimately selected five key features: BNP, lactate, left ventricular ejection fraction (LVEF), troponin, and central venous pressure (CVP). The ISGA convergence curves of the optimal model are shown in Supplementary Figure S3 in Supplementary File 3.

**Table 2:** Performance metrics of prediction models.

Date set	Models	PRE	SEN	SPE	ACC	F1	ROC-AUC	PR-AUC
Training set	LR	0.7153 (0.6619–0.7734)	0.8305 (0.7627–0.8983)	0.6929 (0.6142–0.7717)	0.7592 (0.7061–0.8082)	0.7686 (0.7177–0.8160)	0.8088 (0.7539–0.8637)	0.7709 (0.7116–0.8302)
	SVM	0.7521 (0.6923–0.8158)	0.7458 (0.6695–0.8220)	0.7717 (0.7008–0.8425)	0.7592 (0.7061–0.8122)	0.7489 (0.6903–0.8050)	0.8037 (0.7481–0.8593)	0.8087 (0.7538–0.8636)
	DT	0.8776 (0.8182–0.9362)	0.7288 (0.6441–0.8051)	0.9055 (0.8504–0.9528)	0.8204 (0.7714–0.8653)	0.7963 (0.7343–0.8520)	0.8747 (0.8295–0.9199)	0.8867 (0.8436–0.9298)
	Adaboost	0.8454 (0.7800–0.9101)	0.6949 (0.6102–0.7797)	0.8819 (0.8189–0.9370)	0.7918 (0.7429–0.8408)	0.7628 (0.6986–0.8214)	0.8717 (0.8260–0.9174)	0.8432 (0.7930–0.8934)
	XGBoost	0.8857 (0.8291–0.9400)	0.7881 (0.7119–0.8559)	0.9055 (0.8504–0.9528)	0.8490 (0.8041–0.8898)	0.8341 (0.7798–0.8814)	0.8978 (0.8568–0.9388)	0.8880 (0.8451–0.9309)
	LightGBM	0.9123 (0.8632–0.9612)	0.8814 (0.8220–0.9322)	0.9213 (0.8740–0.9685)	0.9020 (0.8653–0.9388)	0.8966 (0.8546–0.9351)	0.9445 (0.9142–0.9748)	0.9455 (0.9155–0.9755)
Testing set	LR	0.7045 (0.6000–0.8158)	0.6739 (0.5435–0.8043)	0.7797 (0.6780–0.8814)	0.7333 (0.6476–0.8095)	0.6889 (0.5806–0.7865)	0.7635 (0.6693–0.8577)	0.6544 (0.5478–0.7610)
	SVM	0.9500 (0.8421–1.0000)	0.4130 (0.2821–0.5652)	0.9831 (0.9492–1.0000)	0.7333 (0.6667–0.8000)	0.5758 (0.4138–0.7042)	0.7502 (0.6540–0.8464)	0.7393 (0.6416–0.8370)
	DT	0.7647 (0.6389–0.8929)	0.5652 (0.4130–0.7174)	0.8644 (0.7627–0.9492)	0.7333 (0.6571–0.8095)	0.6500 (0.5205–0.7619)	0.7929 (0.7035–0.8823)	0.7599 (0.6652–0.8546)
	Adaboost	0.7308 (0.6406–0.8298)	0.8261 (0.7174–0.9348)	0.7627 (0.6441–0.8644)	0.7905 (0.7143–0.8667)	0.7755 (0.6923–0.8542)	0.8545 (0.7778–0.9312)	0.7751 (0.6827–0.8675)
	XGBoost	0.7143 (0.6308–0.8077)	0.8696 (0.7609–0.9565)	0.7288 (0.6102–0.8305)	0.7905 (0.7143–0.8667)	0.7843 (0.7059–0.8571)	0.8254 (0.7422–0.9086)	0.7163 (0.6157–0.8169)
	LightGBM	0.9706 (0.9070–1.0000)	0.7174 (0.5870–0.8478)	0.9831 (0.9492–1.0000)	0.8667 (0.8095–0.9238)	0.8250 (0.7273–0.9070)	0.9289 (0.8912–0.9587)	0.8892 (0.8421–0.9263)

Values are presented as point estimate (95% confidence interval). All 95% CIs were estimated via bootstrap resampling (1,000 iterations) with the percentile method.

On the independent test set, the AutoML model retained robust performance with a ROC-AUC of 0.9289 and a PR-AUC of 0.8892 (Figures 2C,D), confirming strong generalization and stable predictive capability. DCA revealed substantial net clinical benefit across risk thresholds of 1%–92%, outperforming conventional approaches (Figure 2E). This consistent curve indicated strong generalization and stable predictive capability. Calibration analysis confirmed AutoML model's calibration accuracy, yielding the lowest test set Brier score (0.114) (Figure 2F).

DeLong's test, applied exclusively to ROC-AUC, showed that LightGBM achieved a significantly higher test-set ROC-AUC than each comparator model (vs. LR: Z = 4.30, *P* < 0.001; vs. SVM: Z = 4.55, *P* < 0.001; vs. DT: Z = 3.73, *P* < 0.001; vs. AdaBoost: Z = 2.35, *P* = 0.019; and vs. XGBoost: Z = 3.04, *P* = 0.002). PR-AUC values were reported descriptively with bootstrap-derived 95% confidence intervals, and no formal pairwise hypothesis testing was performed for PR-AUC. The Brier score for LightGBM on the test set was 0.114 (95% CI: 0.091–0.141), indicating good calibration.

### 3.3 Performance of the improved swarm intelligence algorithm and ablation validation

The Improved Snow Geese Algorithm (ISGA) proposed in this study enhanced the optimization capability over SGA through Logistic chaotic initialization (*μ*=4.0) and dynamic Lévy flight (*β*=1.5). To evaluate the actual benefit of ISGA on clinical endpoints, an ablation experiment was conducted on the independent test set (Supplementary File 1 Table S3): with LightGBM as the base learner, four strategies—ISGA-AutoML, Bayesian optimization, random search, and grid search—were respectively employed for joint feature selection and hyperparameter optimization. The results (Supplementary Table S3) showed that ISGA-AutoML achieved the highest test set ROC-AUC (0.9289, 95% CI: 0.8912–0.9587) and PR-AUC (0.8892, 95% CI: 0.8421–0.9263), as well as the lowest Brier score (0.114, 95% CI: 0.091–0.141). The DeLong test confirmed that the difference in test-set ROC-AUC between ISGA and Bayesian optimization was statistically significant (Z = 2.14, *P* = 0.032). Supplementary File 3 Figures S1, S2 provide the optimization performance comparison and convergence curves of ISGA on the CEC2022 benchmark functions.

### 3.4 Sensitivity analysis of missing value handling

The missing value sensitivity analysis showed that when using within-fold single imputation during training, the final model achieved a test set ROC-AUC of 0.9289 (95% CI: 0.8912–0.9587), a PR-AUC of 0.8892 (95% CI: 0.8421–0.9263), and a Brier score of 0.114. The corresponding results for the complete case analysis were 0.9216 (95% CI: 0.8794–0.9542), 0.8765 (95% CI: 0.8237–0.9189), and 0.121, respectively. The corresponding results for the multiple imputation (m = 20) analysis were 0.9312 (95% CI: 0.8935–0.9601), 0.8918 (95% CI: 0.8459–0.9287), and 0.112, respectively. The results of the three handling strategies were consistent in direction and their 95% confidence intervals overlapped substantially, suggesting that the missing value handling approach did not materially affect the model conclusions.

### 3.5 Key influencing factor analysis

#### 3.5.1 LASSO regression

**Figure 3:**
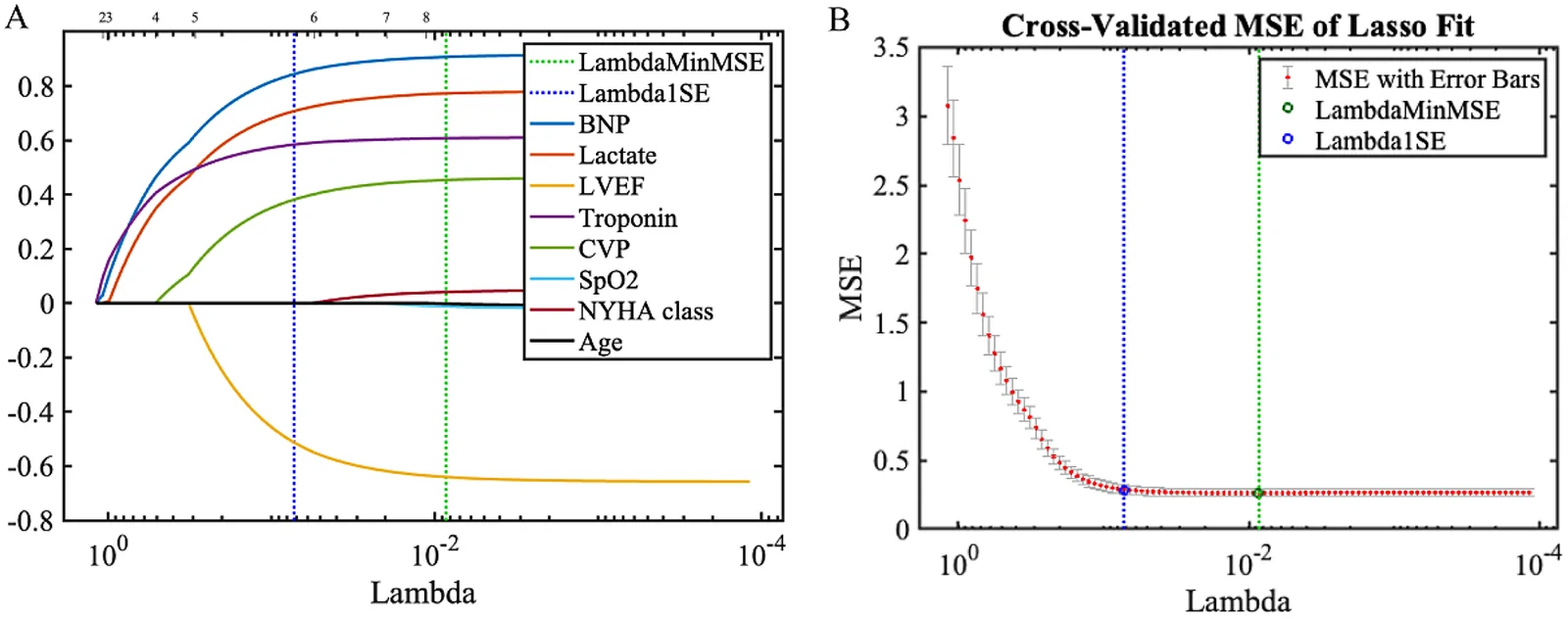
LASSO regression results. **(A)** LASSO solution trajectory; **(B)** Cross-validation error curve.

LASSO regression was applied to the training set for feature screening (Figure 3) to validate the robustness of the AutoML feature selection. Figure 3A illustrates the trajectory of coefficient variation of LASSO across the full regularization path: approximately 8 variables had non-zero coefficients at the minimum *λ*, but as *λ* increased to the optimal value (*λ*_1SE, i.e., the sparsest model within one standard error of the minimum mean squared error), the regularization penalty shrank the coefficients of weaker contributors to zero, ultimately retaining five variables with non-zero coefficients: BNP, lactate, left ventricular ejection fraction (LVEF), troponin, and central venous pressure (CVP). These five variables exactly and completely covered the features selected by the AutoML framework, and the consistency between two independent methods in feature identification reinforced the reliability of the predictors. Figure 3B presents the cross-validation error curve, with the blue vertical line marking the position of *λ*_1SE.

#### 3.5.2 Interpretability analysis

**Figure 4:**
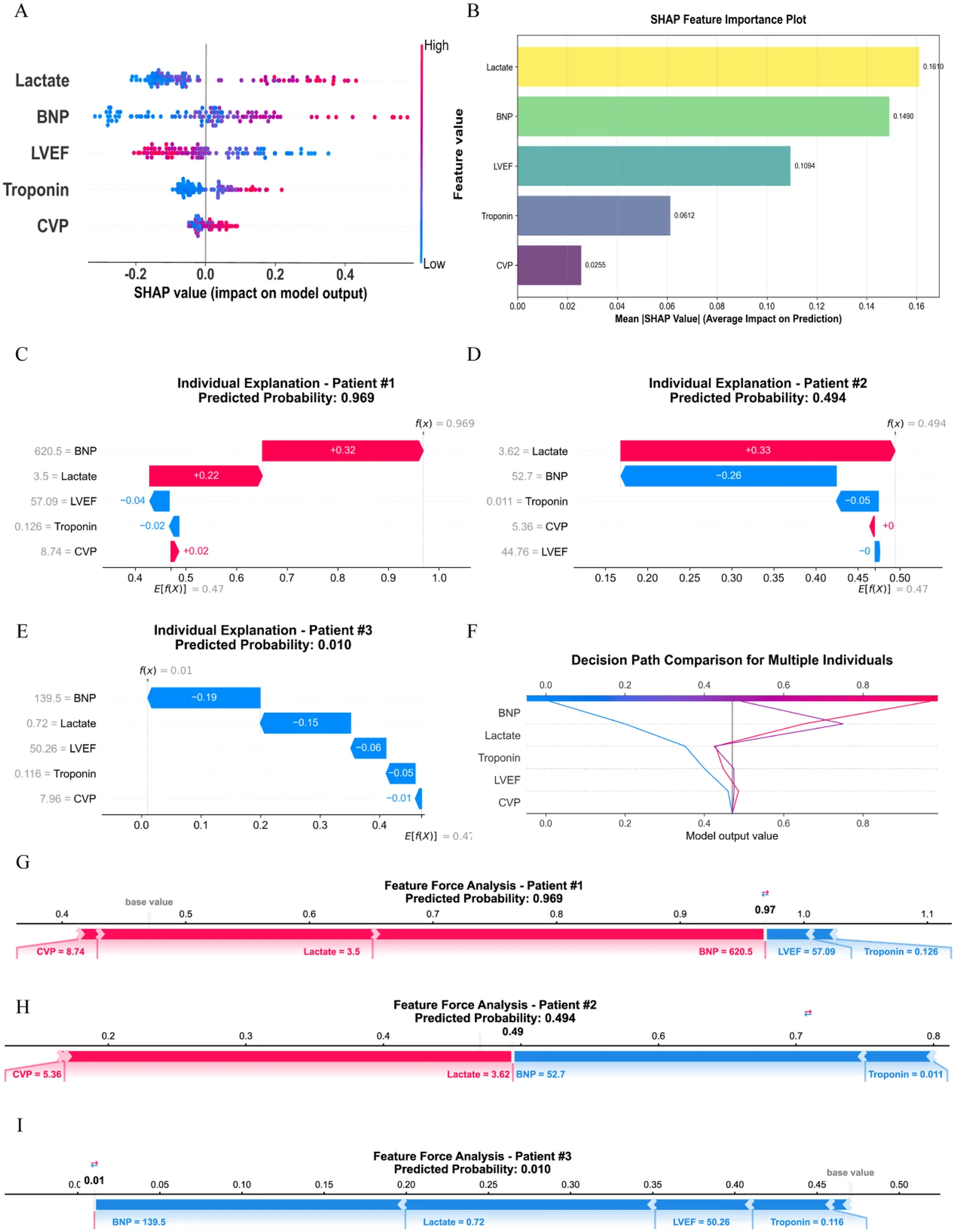
**(A)** Summary plot. **(B)** Feature importance plot. **(C-E)** Waterfall plots showing each feature's contribution to individual predictions. **(F)** Decision path plot comparing decision routes across patients. **(G-I)** Force plots illustrating how features push predictions toward higher (red) or lower (blue) risk.

SHAP analysis ranked feature importance as: lactate > BNP > LVEF > troponin > CVP (Figure 4A,B), aligning with pathological drivers of adverse outcomes. Contrasting decision paths for high-/low-risk patients revealed systematic rightward shifts in high-risk cases due to cumulative adverse features (Figure 4F). Individual interpretations are detailed below:

Case 1 (High Risk, Figures 4C,G): Significantly elevated BNP (620.5 pg/mL) and lactate (3.5 mmol/L) indicated cardiac dysfunction and metabolic disturbance, despite near-normal LVEF (57.09%). The model predicted 96.89% extubation failure probability, validated by actual failure due to dominant feature impacts.
Case 2 (Moderate Risk, Figures 4D,H): Low BNP (52.7 pg/mL), troponin, and CVP suggested stable cardiac function, counterbalanced by elevated lactate (3.62 mmol/L) possibly linked to non-cardiac factors. The moderate risk prediction (49.42% failure probability) reflected model integration capabilities, with successful extubation confirming balanced assessment.
Case 3 (Low Risk, Figures 4E,I): All features (BNP=139.5 pg/mL, lactate=0.72 mmol/L, LVEF=50.26%) were favorable, yielding minimal risk (failure probability=1.02%) and accurate prediction of successful extubation, affirming model reliability in low-risk identification.

### 3.6 Visualization system development

**Figure 5:**
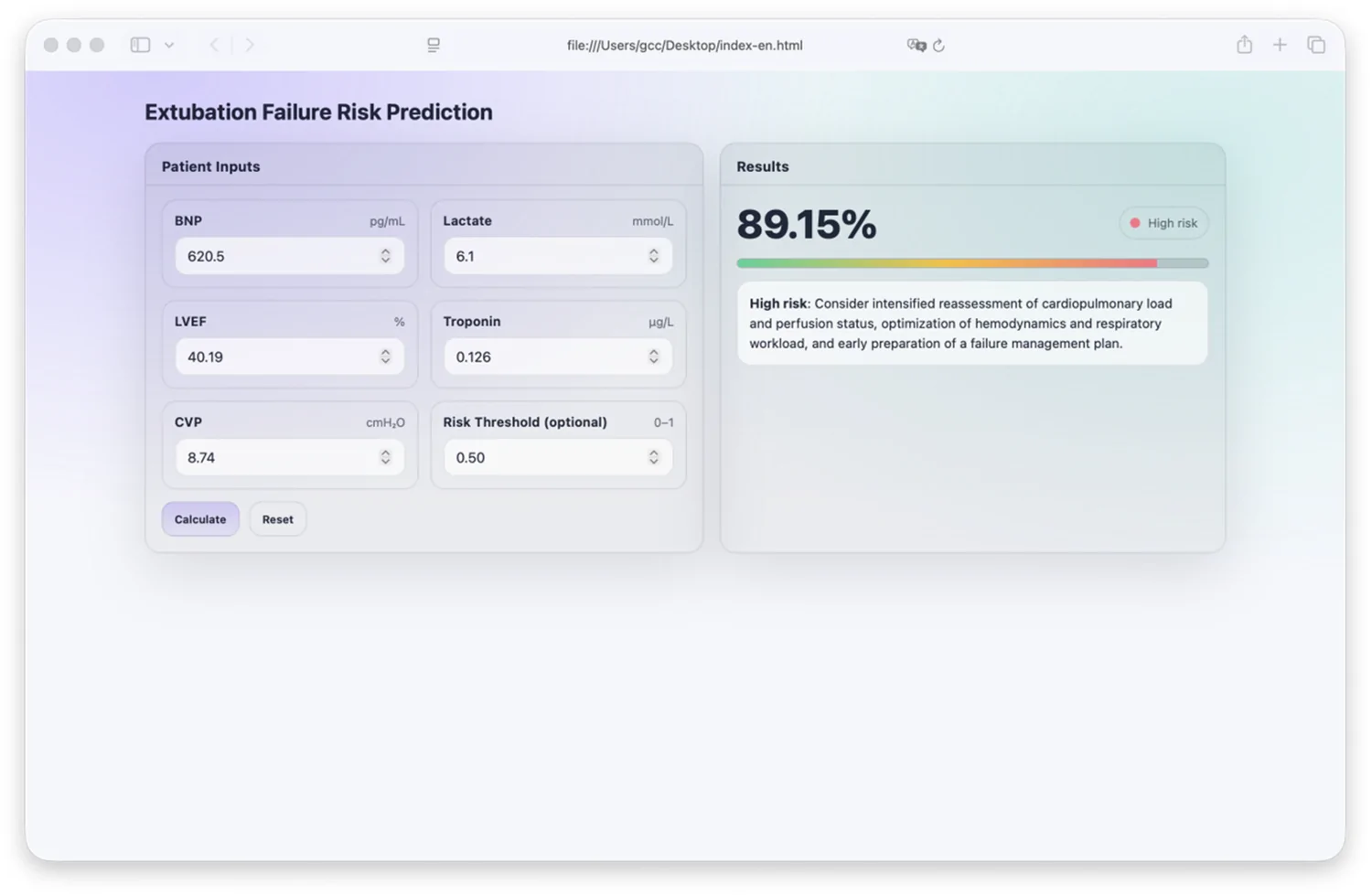
Visualization system for extubation failure risk prediction.

To enable clinical translation, we developed an intuitive visualization system where clinicians need only input the 5 key features to automatically calculate prognosis (Figure 5).

## 4 Discussion

This study establishes a predictive framework for extubation failure in invasively ventilated patients with combined respiratory failure and cardiac dysfunction. We developed a supervised AutoML model for a cohort of 350 patients, incorporating SHAP interpretability analysis. This approach identified five core predictors of extubation failure: B-type natriuretic peptide (BNP), lactate, left ventricular ejection fraction (LVEF), troponin, and central venous pressure (CVP). These pathophysiologically interlinked biomarkers form a targeted decision-support tool for mechanical ventilation management.

The pathophysiological interplay among the five core predictors—BNP, lactate, LVEF, troponin, and CVP—forms a hemodynamically coherent framework for extubation failure prediction in cardiorespiratory dysfunction. BNP elevation not only reflects ventricular wall stress but also amplifies neurohormonal activation (e.g., RAAS/SNS), directly promoting cardiac remodeling, pulmonary vasoconstriction, and alveolar fluid accumulation (23). This process establishes a cardiopulmonary vicious cycle where impaired LVEF (<40%) critically reduces systemic oxygen delivery capacity (24), triggering tissue hypoperfusion manifesting as hyperlactatemia (>4 mmol/L). Such lactate elevation signals profound metabolic dysregulation through both anaerobic glycolysis and mitochondrial dysfunction (25, 26). Concurrently, persistent troponin elevation—even below infarction thresholds—indicates cumulative cardiomyocyte injury from sustained tachycardia, coronary hypoperfusion, and inflammatory toxicity (27, 28), which further diminishes myocardial contractility and promotes arrhythmogenesis. Crucially, elevated CVP (>12 mmHg) intensifies right ventricular afterload, compresses pulmonary microvasculature, and reduces venous return, thereby amplifying ventricular interdependence and worsening gas exchange efficiency (29). Mechanistically, CVP elevation both stimulates compensatory BNP secretion and exacerbates pulmonary edema through increased transcapillary filtration. It should be noted that the thresholds cited in the Discussion—such as LVEF <40%, lactate >4 mmol/L, and CVP >12 mmHg—are all literature-based clinical reference standards for high-risk stratification. These values were introduced solely to facilitate pathophysiological interpretation of the identified predictors and to relate their clinical significance to established evidence, not as classification cut-offs derived from our model. Importantly, the model developed in this study outputs a continuous predicted probability of extubation failure, thereby allowing clinicians to flexibly select decision thresholds tailored to institutional practices and risk tolerance. Additionally, the 48-h composite endpoint of reintubation, unplanned NIV, and death may involve a competing risk structure where early mortality precludes the observation of reintubation. Our conservative approach of attributing indeterminate deaths to extubation failure may slightly overestimate the failure rate; future studies should employ competing-risk regression (e.g., Fine-Gray model) to disentangle these events.

This study's strength lies in its integrated pursuit of “precision prediction, in-depth interpretation, and practical translation” as a tripartite objective. Departing from “black-box” machine learning or conventional regression, our supervised approach emphasizes interpretability through a systematic dual-perspective explainability analysis (SHAP-driven feature prioritization+multivariable regression validation). This “dual-locking strategy”—combining machine learning's capacity for complex pattern recognition with traditional statistics' rigorous validation—ensures that the identified predictors possess clinical relevance, biological plausibility, and statistical robustness. Critically, to bridge the clinical AI adoption gap, we engineered an intuitive visualized decision-support software enabling “a prototype system toward clinical translation”. This paradigm of “discovery-interpretation-translation” represents a key emphasis of the present work, aiming to bridge the gap between model development and potential clinical application.

### 4.1 Limitations and future directions

Despite progress in multidimensional prediction modeling, interpretability analysis, and clinical translation, several limitations persist: (1) Retrospective design: Complete reliance on historical electronic medical records carries risks of information bias and key variable omissions despite structured extraction and verification. Non-randomized treatment decisions introduce unavoidable confounding effects. (2) Single-center constraint: All 350 patients originated from Hangzhou Lin'an District First Hospital, limiting generalizability to diverse hospital levels or geographic settings. (3) Feature gaps: Although broad predictors were included, potentially significant dimensions like unrecorded genetic susceptibility markers, psychosocial factors, and dynamic time-series data were omitted, potentially restricting model performance. (4) The validation of this study was based on a single 70/30 training/test set split. Although supplemented by Bootstrap internal validation (1,000 resampling iterations), it still lacked an independent external validation cohort. Therefore, the generalizability of the model in external populations remains unclear. The currently developed visualization system should be regarded as a prototype tool, and its clinical effectiveness and utility need to be further evaluated through prospective multicenter studies. (5) Missing value handling methods: In this study, the overall missing rate for candidate variables was relatively low, and model performance was largely consistent across different missing value handling strategies. However, the missing-at-random assumption cannot be fully verified through observational data, and residual bias caused by non-random missingness cannot be completely ruled out. The main analysis employed within-fold single imputation during training; although this facilitates model reproducibility and clinical deployment, it cannot fully propagate the uncertainty associated with missing values as multiple imputation does. Therefore, the relevant results still require validation in prospective multicenter cohorts with higher data completeness.

Addressing these limitations necessitates prioritizing: (1) Prospective multi-center validation across diverse institutions and patient cohorts; (2) Dynamic early-warning systems integrating IoT/wearable technologies for real-time feature monitoring & time-series algorithms (e.g., RNN, LSTM) to track “dynamic risk evolution trajectories”; (3) Prospective RCTs to bridge the clinical translation gap and validate real-world impact.

## 5 Summary

In summary, this study demonstrates a promising approach in predicting extubation failure for mechanically ventilated patients with respiratory failure complicated by cardiac dysfunction. We established a prototype predictive model utilizing supervised machine learning, which precisely assesses extubation risk through five core cardiopulmonary parameters: BNP, lactate, LVEF, troponin, and CVP. An innovative dual-explainability pathway integrating SHAP-driven feature interpretation and statistical validation was developed, elucidating pathophysiological interactions among predictors. Crucially, through a feature-based visualized decision support system enabling real-time bedside risk calculation and intervention guidance, we demonstrate a proof-of-concept translation from predictive analytics to clinical implementation. These contributions collectively bridge the gap between computational modeling and critical care practice, advancing precision management paradigms for complex cardiorespiratory failure.

## Data Availability

The raw data supporting the conclusions of this article will be made available by the authors, without undue reservation.
