# Supplementary material for: Predicting extubation failure in patients with cardiorespiratory decompensation: development and validation of an interpretable machine learning–based risk calculator
Source: Front Cardiovasc Med. 2026 Aug 24;13:1862117. doi: 10.3389/fcvm.2026.1862117 (PMC13547519; doi:10.3389/fcvm.2026.1862117)
Supplement: Supplementary file 1 [file Supplementaryfile1.docx]

Table S1. ISGA Parameter Configuration

| Parameter | Value |
| --- | --- |
| Population size | 30 |
| Maximum iterations | 50 |
| Independent runs | 10 |
| Final selection strategy | Best fitness across runs |
| Chaotic map | Logistic map (μ = 4.0) |
| Lévy flight β | 1.5 |
| Lévy step scaling factor | 0.01 × (ub − lb) |
| Dimension encoding | [Feature mask \| Model type \| Hyperparameter vector] |
| Fitness function | 1 − mean 5-fold CV AUC |

Table S2. Hyperparameter Search Spaces and Final Optimal Configurations for All Base Learners

| Base learner | Hyperparameter | Search range | Final value | Type |
| --- | --- | --- | --- | --- |
| LR | Lambda (L2 regularization) | [1e−4, 10] | 0.56 | Continuous |
| SVM | KernelScale | [0.1, 10] | 1.8 | Continuous |
|  | BoxConstraint | [0.1, 10] | 2.5 | Continuous |
| DT | MinLeafSize | [1, 20] | 5 | Integer |
|  | MaxDepth | [3, 15] | 7 | Integer |
| AdaBoost | NumLearningCycles | [10, 200] | 80 | Integer |
|  | LearnRate | [0.01, 1.0] | 0.68 | Continuous |
| XGBoost | MaxDepth | [3, 10] | 6 | Integer |
|  | LearningRate | [0.01, 0.3] | 0.095 | Continuous |
|  | nEstimators | [50, 500] | 200 | Integer |
|  | Subsample | [0.5, 1.0] | 0.78 | Continuous |
|  | ColSampleByTree | [0.5, 1.0] | 0.82 | Continuous |
|  | RegLambda (L2) | [0, 10] | 1.25 | Continuous |
|  | RegAlpha (L1) | [0, 10] | 0.15 | Continuous |
| LightGBM | NumLeaves | [2, 50] | 31 | Integer |
|  | LearningRate | [0.01, 0.3] | 0.072 | Continuous |
|  | MinDataInLeaf | [3, 30] | 12 | Integer |
|  | FeatureFraction | [0.6, 1.0] | 0.85 | Continuous |
|  | BaggingFraction | [0.6, 1.0] | 0.8 | Continuous |
|  | BaggingFreq | [1, 10] | 4 | Integer |
|  | LambdaL1 | [0, 5] | 0.12 | Continuous |
|  | LambdaL2 | [0, 5] | 0.35 | Continuous |
|  | MaxDepth | [3, 15] | 8 | Integer |

Note: LightGBM final values represent the globally optimal configuration selected by the ISGA-AutoML framework across all six base learners.

Table S3. Ablation Study: Comparison of Optimization Strategies on the Clinical Test Set

| Optimization Strategy | Test ROC-AUC (95% CI) | Test PR-AUC (95% CI) | Brier Score (95% CI) |
| --- | --- | --- | --- |
| ISGA-AutoML (Proposed) | 0.9289 (0.8912–0.9587) | 0.8892 (0.8421–0.9263) | 0.114 (0.091–0.141) |
| Bayesian Optimization | 0.9103 (0.8698–0.9441) | 0.8647 (0.8125–0.9078) | 0.128 (0.103–0.157) |
| Random Search | 0.8951 (0.8512–0.9315) | 0.8462 (0.7903–0.8934) | 0.142 (0.115–0.173) |
| Grid Search | 0.8876 (0.8423–0.9258) | 0.8351 (0.7768–0.8842) | 0.151 (0.122–0.184) |

Note: All metrics based on LightGBM base learner. 95% CIs estimated via 1,000 bootstrap iterations. DeLong test for pairwise ROC-AUC comparison: ISGA vs. Bayesian Optimization, Z=2.14, P=0.032; ISGA vs. Random Search, Z=2.89, P=0.004; ISGA vs. Grid Search, Z=3.21, P=0.001.
