# Supplementary material for: Predicting extubation failure in patients with cardiorespiratory decompensation: development and validation of an interpretable machine learning–based risk calculator
Source: Front Cardiovasc Med. 2026 Aug 24;13:1862117. doi: 10.3389/fcvm.2026.1862117 (PMC13547519; doi:10.3389/fcvm.2026.1862117)
Supplement: Supplementary file 2 [file Supplementaryfile2.docx]

**ISGA-AutoML: Synchronous Feature Selection, Model Selection, and Hyperparameter Optimization via Improved Snow Geese Algorithm**

%% ===== Input =====

% X_train, y_train : Training data

% nFeatures : Number of candidate features

% modelHPBounds : Struct array (length 6) with HP bounds for

% each base learner (1:LR, 2:SVM, 3:DT,4:AdaBoost, 5:XGBoost, 6:LightGBM)

% maxHPDim : Maximum HP dimension across all learners

%% ===== Step 0: Parameter Initialization =====

popSize = 30; % Population size

maxIter = 50; % Maximum iterations

dim = nFeatures + 1 + maxHPDim; % [featMask | modelType | HPvec]

lb = [zeros(1, nFeatures), 1, modelHPBounds.lb];

ub = [ones(1, nFeatures), 6.5, modelHPBounds.ub];

%% ===== Step 1: Chaotic Initialization (Logistic map) =====

pop = zeros(popSize, dim);

seed = rand(1, dim);

for i = 1:popSize

seed = 4.0 * seed .* (1 - seed); % x_{n+1} = μ·x_n·(1-x_n)

pop(i, :) = seed;

end

pop = pop .* (ub - lb) + lb;

for i = 1:popSize

fitness(i) = evaluate(pop(i, :), X_train, y_train);

end

[~, bestIdx] = min(fitness);

gbest = pop(bestIdx, :);

gbestFit = fitness(bestIdx);

%% ===== Step 2: Main Optimization Loop =====

for t = 1:maxIter

for i = 1:popSize

%% --- Lévy Flight ---

beta = 1.5;

sigma = (gamma(1+beta)*sin(pi*beta/2) / ...

(gamma((1+beta)/2)*beta*2^((beta-1)/2)))^(1/beta);

u = normrnd(0, sigma);

v = normrnd(0, 1);

stepSize = 0.01 * (ub - lb) .* (u ./ abs(v)^(1/beta));

%% --- SGA Position Update ---

if rand < 0.5 % Exploration

rIdx = randi(popSize);

newPos = pop(i,:) + stepSize .* (pop(rIdx,:) - pop(i,:));

else % Exploitation

newPos = pop(i,:) + stepSize .* (gbest - pop(i,:));

end

newPos = max(min(newPos, ub), lb); % Boundary clamp

%% --- Fitness Evaluation ---

newFit = evaluate(newPos, X_train, y_train);

%% --- Greedy Selection ---

if newFit < fitness(i)

pop(i,:) = newPos;

fitness(i) = newFit;

end

end

[minFit, bestIdx] = min(fitness);

if minFit < gbestFit

gbest = pop(bestIdx,:);

gbestFit = minFit;

end

end

%% ===== Step 3: Extract Optimal Configuration =====

featMask = round(gbest(1:nFeatures));

selectedFeatures = find(featMask);

modelType = round(gbest(nFeatures + 1));

optimalHP = gbest(nFeatures + 2 : end);

%% ===== Evaluate: 5-fold CV AUC =====

function loss = evaluate(position, X, y)

featMask = round(position(1:nFeatures));

modelType = round(position(nFeatures + 1));

hpVector = position(nFeatures + 2 : end);

X_sub = X(:, featMask == 1);

switch modelType

case 1 % Logistic Regression

hp = struct('Lambda', hpVector(1));

model = fitclinear(X_sub, y, 'Learner', 'logistic', ...

'Lambda', hp.Lambda, 'CV', 'on', 'KFold', 5);

case 2 % SVM

hp = struct('KernelScale', hpVector(1), ...

'BoxConstraint', hpVector(2));

model = fitcsvm(X_sub, y, 'KernelFunction', 'rbf', ...

'KernelScale', hp.KernelScale, ...

'BoxConstraint', hp.BoxConstraint, ...

'CrossVal', 'on', 'KFold', 5);

case 3 % Decision Tree

hp = struct('MinLeafSize', round(hpVector(1)), ...

'MaxDepth', round(hpVector(2)));

model = fitctree(X_sub, y, 'MinLeafSize', hp.MinLeafSize, ...

'MaxDepth', hp.MaxDepth, 'CrossVal', 'on');

case 4 % AdaBoost

hp = struct('NLearn', round(hpVector(1)), ...

'LearnRate', hpVector(2));

model = fitcensemble(X_sub, y, 'Method', 'AdaBoostM2', ...

'NumLearningCycles', hp.NLearn, ...

'LearnRate', hp.LearnRate, 'CrossVal', 'on');

case 5 % XGBoost

hp = struct('MaxDepth', round(hpVector(1)), ...

'LearningRate', hpVector(2), ...

'Subsample', hpVector(3), ...

'ColSample', hpVector(4), ...

'RegLambda', hpVector(5));

model = fit_xgboost_cv(X_sub, y, hp, 'KFold', 5);

case 6 % LightGBM

hp = struct('NumLeaves', round(hpVector(1)), ...

'LearningRate', hpVector(2), ...

'MinDataInLeaf', round(hpVector(3)), ...

'FeatureFraction', hpVector(4), ...

'BaggingFraction', hpVector(5), ...

'LambdaL1', hpVector(6), 'LambdaL2', hpVector(7));

model = fit_lightgbm_cv(X_sub, y, hp, 'KFold', 5);

end

[~, scores] = kfoldPredict(model);

[~, ~, ~, AUC] = perfcurve(y, scores(:, 2), '1');

loss = 1 - AUC; % Minimize

end
