# Supplementary material for: Predicting extubation failure in patients with cardiorespiratory decompensation: development and validation of an interpretable machine learning–based risk calculator
Source: Front Cardiovasc Med. 2026 Aug 24;13:1862117. doi: 10.3389/fcvm.2026.1862117 (PMC13547519; doi:10.3389/fcvm.2026.1862117)
Supplement: Supplementary file 3 [file Supplementaryfile3.docx]

**1-Improved Swarm Intelligence Algorithm Performance**

Validation results demonstrate the ISGA's superior optimization capability, outperforming baseline SGA and other algorithms (PSO, HHO, WOA) across most CEC2022 benchmark functions with enhanced stability (**Fig. S1**). Convergence curve analysis confirms ISGA achieves faster convergence rates and minimal susceptibility to local optima during iterations (**Fig. S2**). These findings validate ISGA's advantages in global optimization and convergence efficiency as the core algorithm for this study.

**Fig. S1 Comparative Optimization Performance of Swarm Intelligence Algorithms**

**Fig. S2 Comparative Convergence Performance**

**2-Convergence trajectory of ISGA on the clinical dataset**

The fitness curve (1 − mean 5-fold CV ROC-AUC) over 50 iterations for the optimal run shows rapid descent during the first 25 iterations (from approximately 0.26 to 0.072), followed by stable convergence. The final best fitness value reached 0.062, corresponding to a mean CV AUC of 0.938. The trajectory confirms that 50 iterations were sufficient for algorithm convergence on the clinical prediction task.

**Figure S3. Convergence trajectory of ISGA on the clinical dataset.**
